# Development of an environmental health tool linking chemical exposures, physical location and lung function

**DOI:** 10.1186/s12889-019-7217-z

**Published:** 2019-07-01

**Authors:** Diana Rohlman, Holly M. Dixon, Laurel Kincl, Andrew Larkin, Richard Evoy, Michael Barton, Aaron Phillips, Elena Peterson, Christopher Scaffidi, Julie B. Herbstman, Katrina M. Waters, Kim A. Anderson

**Affiliations:** 1 College of Public Health and Human Sciences; Superfund Research Program, Oregon State University, 101 Milam Hall, Corvallis, Oregon USA; 2 Environmental and Molecular Toxicology, Food Safety and Environmental Stewardship Program, Oregon State University, Corvallis, Oregon USA; 3 College of Public Health and Human Sciences, Oregon State University, Corvallis, Oregon USA; 4 Superfund Research Program, Food Safety and Environmental Stewardship Program, Oregon State University, Corvallis, Oregon USA; 5 Computing & Analytics Division, Pacific Northwest National Laboratory, Richland, Washington USA; 6 College of Engineering, Oregon State University, Corvallis, Oregon USA; 7 Environmental Health Sciences, Mailman School of Public Health, Columbia University, New York City, USA; 8 Biological Sciences Division, Pacific Northwest National Laboratory, Pacific Northwest National Laboratory, Richland, WA USA

**Keywords:** Environmental health, PAHs, Asthma, Air quality, Wearable sensors, Exposome, Silicone wristbands, Spirometer

## Abstract

**Background:**

A challenge in environmental health research is collecting robust data sets to facilitate comparisons between personal chemical exposures, the environment and health outcomes. To address this challenge, the Exposure, Location and lung Function (ELF) tool was designed in collaboration with communities that share environmental health concerns. These concerns centered on respiratory health and ambient air quality. The ELF collects exposure to polycyclic aromatic hydrocarbons (PAHs), given their association with diminished lung function. Here, we describe the ELF as a novel environmental health assessment tool.

**Methods:**

The ELF tool collects chemical exposure for 62 PAHs using passive sampling silicone wristbands, geospatial location data and respiratory lung function measures using a paired hand-held spirometer. The ELF was tested by 10 individuals with mild to moderate asthma for 7 days. Participants wore a wristband each day to collect PAH exposure, carried a cell phone, and performed spirometry daily to collect respiratory health measures. Location data was gathered using the geospatial positioning system technology in an Android cell-phone.

**Results:**

We detected and quantified 31 PAHs across the study population. PAH exposure data showed spatial and temporal sensitivity within and between participants. Location data was used with existing datasets such as the Toxics Release Inventory and the National Oceanic and Atmospheric Administration (NOAA) Hazard Mapping System. Respiratory health outcomes were validated using criteria from the American Thoracic Society with 94% of participant data meeting standards. Finally, the ELF was used with a high degree of compliance (> 90%) by community members.

**Conclusions:**

The ELF is a novel environmental health assessment tool that allows for personal data collection spanning chemical exposures, location and lung function measures as well as self-reported information.

**Electronic supplementary material:**

The online version of this article (10.1186/s12889-019-7217-z) contains supplementary material, which is available to authorized users.

## Background

The Exposure, Location and lung Function (ELF) tool (previously called the Mobile Exposure Device) is an example of an innovative approach to community-defined research needs [1]. As described previously [1], the ELF combines a portable spirometer, a customizable app (ELF Tracker) on an Android smart phone [2] and lightweight silicone wristbands [3]. This allows the ELF to simultaneously record daily chemical exposure for polycyclic aromatic hydrocarbons (PAHs), geospatial coordinates of participants, and lung function measurements.

There has been an increasing need for personal chemical monitoring devices that are low-cost, easy to use, require minimal maintenance and can generate robust, reliable data [4–7]. Current personal chemical exposure monitors are often hampered by a limited range of chemical substrates detected, a need for power (electrical or battery) and maintenance, and can be bulky,difficult to use and may alter a participant’s behavior due to the weight (~5lbs) [8–10]. In addition, the need to evaluate chemicals as complex mixtures rather than individually, has added a difficult layer for personal monitoring. Studies have shown that passive samplers reflect the bioavailable fraction of lipophilic organic chemicals [11, 12]. When tested concurrently with an active air monitor backpack (capable of detecting both semi-volatile and particulate matter), the wristband correlated more strongly with urinary PAH metabolites than either the polyurethane foam or filter [10]. Similarly, other studies report strong significant correlations between concentrations in wristbands and concentrations in urine for flame retardants [13, 14] and nicotine [15]. Furthermore, the passive wristband sampler has high temporal and spatial sensitivity [3], and, to date, has been used to detect and quantify 1530 different organic chemicals, including 62 different PAHs [3, 10, 16, 17]. PAHs are present in crude oil, tobacco smoke, certain petroleum products and are produced through incomplete combustion, such as the burning of fuel or smoking/charbroiling food [18]. Exposure to PAHs has been linked with diminished respiratory health [19–26].

Mobile phones have the ability to track geospatial location and applications (apps) can include questionnaires to add personal reporting around environmental monitoring [27–30]. Recently, the Smoke Sense app released by the Environmental Protection Agency demonstrated the integration of self-reported health data with exposure to smoke from wildfires [31]. The use of apps for disease management has also been explored, such as colorimetric tests for detecting glucose, protein and pH levels in urine [32], as well as cell phones for collecting basic scientific information [33]. Other apps have been developed to integrate with external instrumentation, such as a lens to collect digital retinal images [34]. Similarly, the Air-Smart Spirometer collects respiratory health measures via an external spirometer with results displayed on a smartphone or tablet [35]. Here, we describe the ELF Tracker which integrates compliance data, collection of location data and collection and transfer of spirometry data via an external spirometer linked via Bluetooth.

Spirometry collects three measures of lung health: forced expiratory volume in 1 s (FEV1), forced vital capacity (FVC) and peak expiratory flow (PEF) [36]. These measures can reflect respiratory responses to exposure to air pollution [37]. Acute exposures (< 24 h) can result in changes to respiratory function measureable via spirometry [37]. Prior studies have successfully used FEV1, FVC and PEF to monitor changes in respiratory function [37–40]. Hand-held spirometers are capable of collecting valid spirometry data outside of a clinical setting [35, 41, 42], making them ideal for multi-day research studies.

The ELF was developed and refined in collaboration with two community groups with similar air quality concerns [1]. In each community, researchers worked with established community groups and built off community-led research initiatives. In Eugene, OR, and Carroll County, OH, residents face concerns from industrial air emissions and emissions from unconventional natural gas drilling, respectively [1]. The ELF was designed to capture the breadth of exposures in a full day (24 h). Community members cited differing schedules and routines as a reason for looking at a full 7-day week, explaining that each day might represent different exposures [1]. In collaboration with these communities, a multidisciplinary team of chemists, software engineers, toxicologists, and environmental public health scientists developed, refined and tested the ELF. To ensure the ELF was responsive to community needs, members in each community tested the ELF, thereby improving the usability and accessibility [1]. The study presented herein further evaluates the ELF to determine feasibility as an environmental health tool.

## Methods

### The ELF tool

**Fig. 1:**
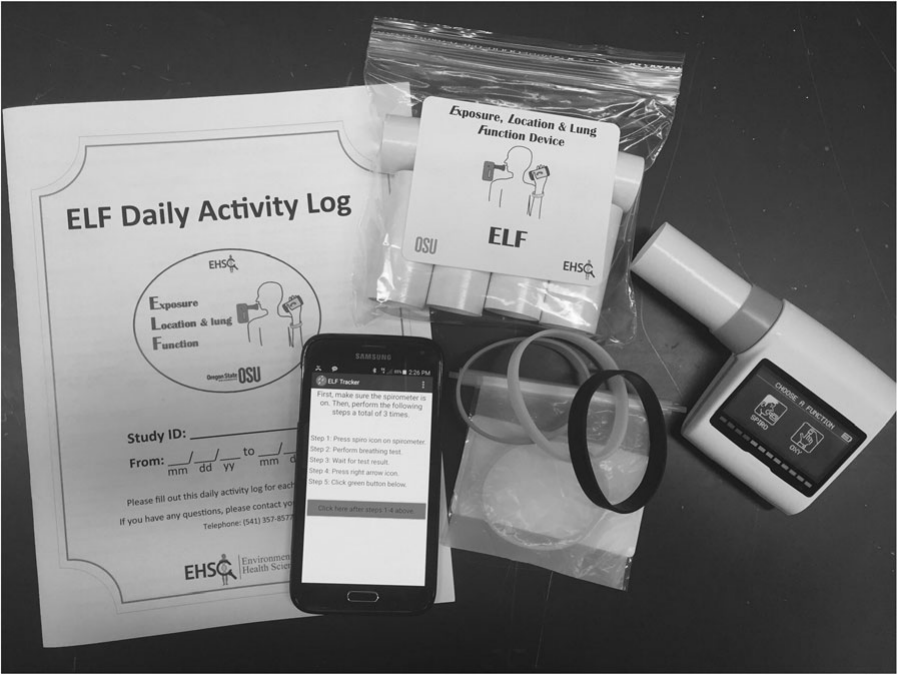
Photograph of the ELF components. Clockwise from left: Activity log for self-reported environmental exposures, disposable paper mouthpieces for use with the hand-held spirometer, 7 silicone wristbands in air-tight packaging, and an Android phone hosting the ELF Tracker

Shown in Fig. 1, the ELF is comprised of a portable spirometer, wristbands, and an Android phone hosting the ELF Tracker stored in a small, shoe-box sized clear traveling case. The portable components of the ELF weigh less than 0.6 pounds and can be easily carried throughout the day. Each ELF component is further described below.

#### Silicone wristbands

Wristbands have been previously described [3, 16]. Each wristband was individually packaged in air-tight polytetrafluoroethylene (PTFE) bags (Welch Fluorocarbon, Dover, NH) for transport to and from participants. Labels were affixed to PTFE bags and participants recorded the date and time they began wearing the wristband as well as when they removed the wristband. Returned wristbands were assessed to ensure they followed the protocol: *i*. worn for 24 h ± 8 h; *ii*. on and off dates/times recorded; *iii*. Worn individually and during appropriate study times and days and; *iv*. placed in a PTFE bag with an air-tight seal.

#### ELF Tracker

The application has been previously described [1, 2]. The ELF Tracker was installed on an Android device with Bluetooth and Global System for Mobile communication (GSM) networking capabilities. The ELF Tracker was designed to transmit continuous spatial location measurements collected from the cell phone, to record and transmit spirometry data, and to prompt participants to complete short questionnaires following each spirometry session. It was pre-loaded with the participant identifier and identifiers for each of the daily wristbands. For each spirometry session, the ELF Tracker asked a series of questions to gauge protocol compliance and self-reported asthma symptoms. Based on community feedback, the ELF Tracker was updated prior to pilot testing to improve usability [1]. The ELF Tracker utilizes data management systems at the Pacific Northwest National Laboratory (PNNL).

#### Portable spirometer

The Spirotel® spirometer (Medical International Research, eHealth minilab, v1) measures lung function via three measurements: FEV_1_, FVC and PEF. Prior to deployment and immediately following collection of the ELF from a study participant, the spirometer was calibrated by measuring three tests with a 3 L calibration syringe (Hans Rudolph Inc., Kansas City, MO). Using the American Thoracic Society (ATS) guidelines, these repeated, controlled volume measures demonstrated that all spirometers were within the acceptable range of +/− 0.15 L (2.85–3.15 L) [36]. The spirometer collects lung function data during a minimum six second test where the user inhales and then exhales forcefully. The spirometer indicates when the test has been completed, which is when at least 6 s have passed.

#### Daily activity log

Each participant was asked to record [yes/no] any use of or exposure to, the following: candles/incense, burnt food, use of a fryer, broiler or charcoal grill and wood-fired heating sources, as well as specific household products known to contain PAHs (i.e. caulks/sealants, spray lubricant, moth balls/flakes, gasoline or vehicle exhaust).

#### Data management system

Study data were collected and stored in a commercial Laboratory Information Management System (LIMS) managed at Oregon State University. These data included participant ID as well as unique identifiers for each wristband in the study. Data generated in the ELF Tracker or by the spirometer was pushed to servers at PNNL through a REST API (Representational State Transfer Application Programming Interface; a common communication protocol in web-based applications). Data was displayed in real-time via a secure, web-based researcher portal. The portal supported input of participant demographic data, ongoing participant monitoring and visualization of exposure, location and lung function data. PNNL servers concurrently received data from the LIMS and the ELF Tracker during the study through secure communication channels, using standard encryption protocols. The password-protected servers are responsible for securely storing and presenting the data as well as performing consistency checks. Constant backups of the data streams are archived at PNNL on secure, password-protected servers. All PNNL servers sit on closely monitored, dedicated, segmented networks, with strict network firewall protection.

### Feasibility study

All activities were conducted under Institutional Review Board approval from Oregon State University (protocol #5736, 8058). Working with a local allergy clinic, eligible participants were contacted via telephone and informed of the study purpose, design and eligibility requirements. Interested participants were asked to contact the research team to determine if they met eligibility requirements: *i*) age 18 or older; *ii*) current asthma diagnosis, *iii*) mild to moderate asthma (assessed via specific questions to gauge asthma severity); *iv*) current non-smoker (prior smoking history allowable) and *v*) live within a 20 mile radius of Eugene, OR. Between August and September 2015, ten participants were enrolled. Each participant met with a member of the research team to discuss the study and answer any questions. Upon verbally indicating they were willing to participate, and understood the research goal and associated activities, each participant signed a written consent form prior to undertaking any study activities.

### Participant training/protocol

The study protocol involved a seven-day data collection period. Participants completed a demographic and respiratory health questionnaire and were instructed in the use of the ELF. Participant training lasted a minimum of 45 min. The NIOSH-certified trainer provided verbal instruction in the use of the spirometer, and coached the participant with the spirometer until a valid reading was obtained. A User Guide was also provided to each participant, along with a one-page abbreviated sheet of instructions. Every 24 h, the daily wristband was removed, sealed in a PTFE bag and replaced with a new wristband. The participant was asked to carry the cell phone, which catalogued and transmitted location and spirometry data to the research team in real-time [1]. Three times a day (morning, noon, evening), participants were asked to perform spirometry readings in triplicate. In the evenings, the ELF Tracker asked questions to capture compliance with the study protocol.

### Evaluation

#### Participant feedback

Participants were asked to complete a short telephone interview regarding their experience with the ELF. Fourteen questions were asked, ranging from ease of use to self-reported compliance and any open-ended feedback from the participant.

#### Compliance and feasibility

The ELF was designed to meet the needs of researchers and community members. Several metrics were identified prior to testing: (1) ability to detect PAHs in a 24-h deployment period with temporal and spatial sensitivity across individuals (Exposure); (2) ability to collect geospatial location and evaluate exposure to the environment (e.g. toxic release industry sites, green spaces, exposure to indoor air pollutants) (Location); (3) reliability of the portable spirometer to collect robust data and identify changes in lung function (lung Function); (4) dependability of data transfer within the ELF and from the ELF to computer servers for secure storage and (5) ability of participants to utilize the device for data collection with a high degree of compliance. While this study was not designed to make inferences regarding health status, examples of how the data can be integrated are shown as appropriate.

### Data analysis

#### Demographic/respiratory Health questionnaire/daily activity log

Questionnaires were transcribed and input into a database. All analyses from the questionnaire data involved descriptive statistics such as computing simple averages and proportions of the study population (i.e. 100% of pilot participants used medication to manage their asthma) using Excel. Data from the Daily Activity Log were similarly analyzed.

#### Spirometry analysis

Upon completion of the study, raw data were analyzed for basic quality control using American Thoracic Society protocols [36] for FEV_1_ analysis. Following quality control of the data, the largest FEV_1_ value from each reading was used for analysis. The total number of valid FEV_1_ readings was calculated to determine the percent compliance with the spirometry protocol.

To compare changes in lung function across participants, the percent predicted FEV_1_ value was calculated. This value adjusts for age, gender, height and ethnicity [43]. This value tracks changes in lung function across participants by comparing the test value FEV_1_ (FEV1_test_) with the value predicted (FEV1_predicted_) for an individual of that gender, age, ethnicity and height: (FEV_1 test_ / FEV_1 predicted_) × 100 = % predicted FEV_1_). The Hankinson 1999 spirometry references values, preferred by NHANES III, were used for all calculations [44, 45].

#### ELF geospatial analysis

Outdoor air pollutant and built environment exposures were estimated by integrating air emission records and remote sensing imagery with participant time-activity patterns using GPS coordinates. Spatial exposure estimates for each GPS coordinate were calculated using the Python version 2.7 background processing extension in the spatial software ArcGIS version 10.3.1. Time-weighted daily averages of spatial exposure estimates were calculated using the statistics software R version 3.4.1. Scripts and synthetic example data are openly available [46]. Information for each environmental database, source and website used in this study are available in Additional file 1: Table S1. Maps throughout this manuscript were created using ArcGIS® software by ESRI©. ArcGIS® and ArcMap™ are the intellectual property of ESRI and are used herein under said license.

##### Air pollutant exposures

Daily and weekly PM_2.5_ exposure estimates were derived by taking the time-weighted average of hourly PM_2.5_ measurements from the nearest EPA monitor [47] for each GPS coordinate (SI Eq. 1). Daily and weekly exposure to toxic release inventory (TRI) emissions were estimated by calculating the time- and inverse-distance weighted TRI annual emissions (SI Eq. 2). Daily and weekly exposure to highway and expressway roads (highly correlated with traffic-related air pollution) were derived by calculating the time-weighted length of roads within a 100 m buffer of each GPS coordinate (SI Eq. 3). Chronic ambient PM_2.5_ and NO_2_ exposure estimates were derived by extracting values at residential locations from annual satellite-based land use regression [48] and geographically-weighted regression [49] models for NO_2_ (100 m resolution) and PM_2.5_ (1 km resolution), respectively.

##### Organic matter exposures

The National Land Cover Classification Database is a classification of all vegetation land cover in the continental US at 30 m resolution. We derived weekly estimates of exposure to multiple vegetation types by summing the element wise-multiplication of the normalized difference vegetation index (NDVI) with binary classifications of land types within 250 m of each GPS coordinate and calculating the time-weighted average (SI Eq. 4). Example vegetation types relevant to respiratory outcomes include hay, grass, trees, and wetlands.

##### Wildfire proximity and smoke density/exposure

Daily and weekly exposure to wildfire smoke were calculated by taking the nearest smoke density measurement for each GPS coordinate (Additional file 1: Table S1). Distance to nearest active wildfire was calculated for each GPS coordinate as well.

#### Silicone wristband analysis

Following deployment, wristbands were stored in amber jars at − 20 °C until extraction. Wristbands were extracted as described previously [3]. Briefly, each wristband was extracted twice in ethyl acetate at room temperature and quantitatively concentrated using TurboVap® evaporators (Biotage LLC, Charlotte, NC). Wristband extracts were quantitatively analyzed for 62 polycyclic aromatic hydrocarbons (PAHs) with an Agilent 7889A gas chromatograph interfaced with an Agilent 7000 MS/MS, as described in Anderson et al. 2015 [50]. A complete list of PAHs and instrumental limits of detections (LODs) are in Additional file 1: Table S2. These 62 PAHs include a wide breadth of physical-chemical properties, ranging from naphthalene’s molecular weight of 128.17 g/mol to dibenzo[a,l]pyrene’s molecular weight of 302.37 g/mol [50]. All chemical data analyses were performed using JMP Pro version 13.0.0. To calculate median PAH concentrations (ng wristband^− 1^), values below the LOD were assigned values equal to one-half the LOD. All concentrations were background and surrogate-corrected.

Wristband quality control (QC) samples represent 49% of all samples analyzed. Blank wristbands were collected during wristband preparation deployment and retrieval. In blank QC samples, 56 of the 62 PAHs were below the LOD. Continuing calibration verifications (CCVs) were analyzed every 10 samples. The CCVs include all target PAHs and, per our data quality objectives, at least 80% of the PAHs in the CCVs were within 20% of the true concentration.

## Results

### Participants

The majority of individuals were female (90%, Table 1), and had associated allergic diseases (hay fever, eczema and allergies), along with a family history of allergies. Participants were majority Caucasian and half the study group were former smokers; all were under medical management for their asthma (Table 1). On average participants spent up to 20% of their time outdoors (SD = 0.16), reporting the rest of the time (80%) spent indoors at home, work, school or transit.

**Table 1:** Participant demographics

Number of participants	10
Average age (years ± SD)	49.1 (14.2)
Gender	
Male	1
Female	9
Employed (%)	90%
Race	
White	9
Asian	1
Other	–
Average age of Asthma Diagnosis (years ± SD)	10.2 (13.7)
Asthma controlled by medication (%)	100%
Former smoker	50%
Associated allergic diagnosis	N (%)
Hay fever diagnosis	6 (60%)
Eczema diagnosis	4 (40%)
Allergies	9 (90%
Specific allergies to plants (hay, trees, grass, pollen)	5 (50%)
Specific allergies to animals	6 (60%)
Family history of allergies	9 (90%)

### ELF feasibility metrics

#### 1. Ability to detect PAHs in a 24-h period with temporal and spatial sensitivity across individuals (exposure)

Of 70 deployed wristbands (10 participants, 7 daily wristbands), 69 were returned, with one wristband lost by a participant. Seven other wristbands did not meet compliance protocols, primarily due to wristbands being worn outside of the designated 24 ± 8 h window (6/7 non-compliant wristbands). The remaining 62 wristbands were used for all further analyses.

**Fig. 2:**
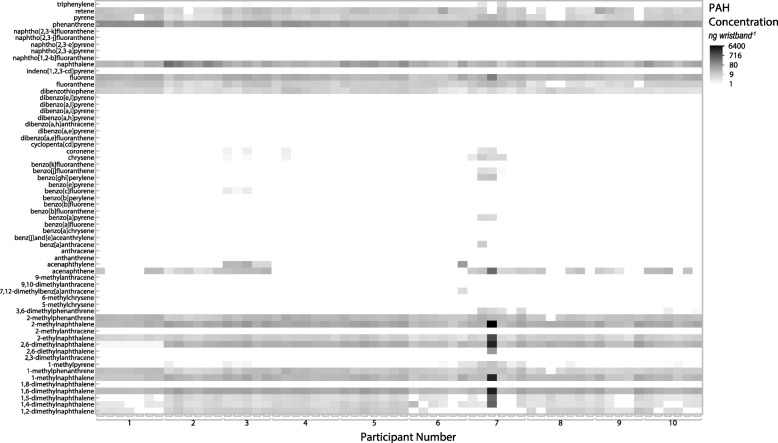
PAH concentrations for the 62 wristbands worn by 10 study participants. The grayscale indicates concentration (on a log scale) for all PAHs detected above the limit of detection (LOD)

Thirty-one PAHs were detected in the wristbands. Nine PAHs were found in all wristbands, (phenanthrene, 2-methylnaphthalene, 1,6-dimethylnaphthalene, naphthalene, 2,6-dimethylnaphthalene, fluorene, 1-methylnaphthalene, 2-ethylnaphthalene, and dibenzothiophene; listed from highest to lowest median concentration) (Additional file 1: Table S2). The median number of PAHs detected per wristband was 17, ranging from 12 to 25 detections. The PAH exposure profile was unique to each individual (Fig. 2).

Participants self-reported a range of exposures to PAH-containing sources. On average, participants reported being exposed to 4 sources: cosmetics/lotions, household cleaners, hair spray and vehicle exhaust.

#### 2. Ability to collect geospatial location and evaluate spatial exposure to the environment

The ELF Tracker utilized a set location sampling schedule (every 15 min) and used estimates from the accelerometer in the Android phone to increase the sampling rate as needed. A total of 9811 GPS coordinates were collected for all 10 participants over 7 days. The average number of GPS coordinates collected per person was 981 (max = 2271, min = 603). Average environmental exposure statistics based on location were calculated on a daily (24-h) basis (Additional file 1: Table S3). For example, data from the ELF Tracker was capable of determining if a participant was driving, as well as calculating the distance from the nearest Toxic Release Inventory source. The location data allows analysis of individual exposures, as well as analysis of correlations between exposure variables.

**Fig. 3:**
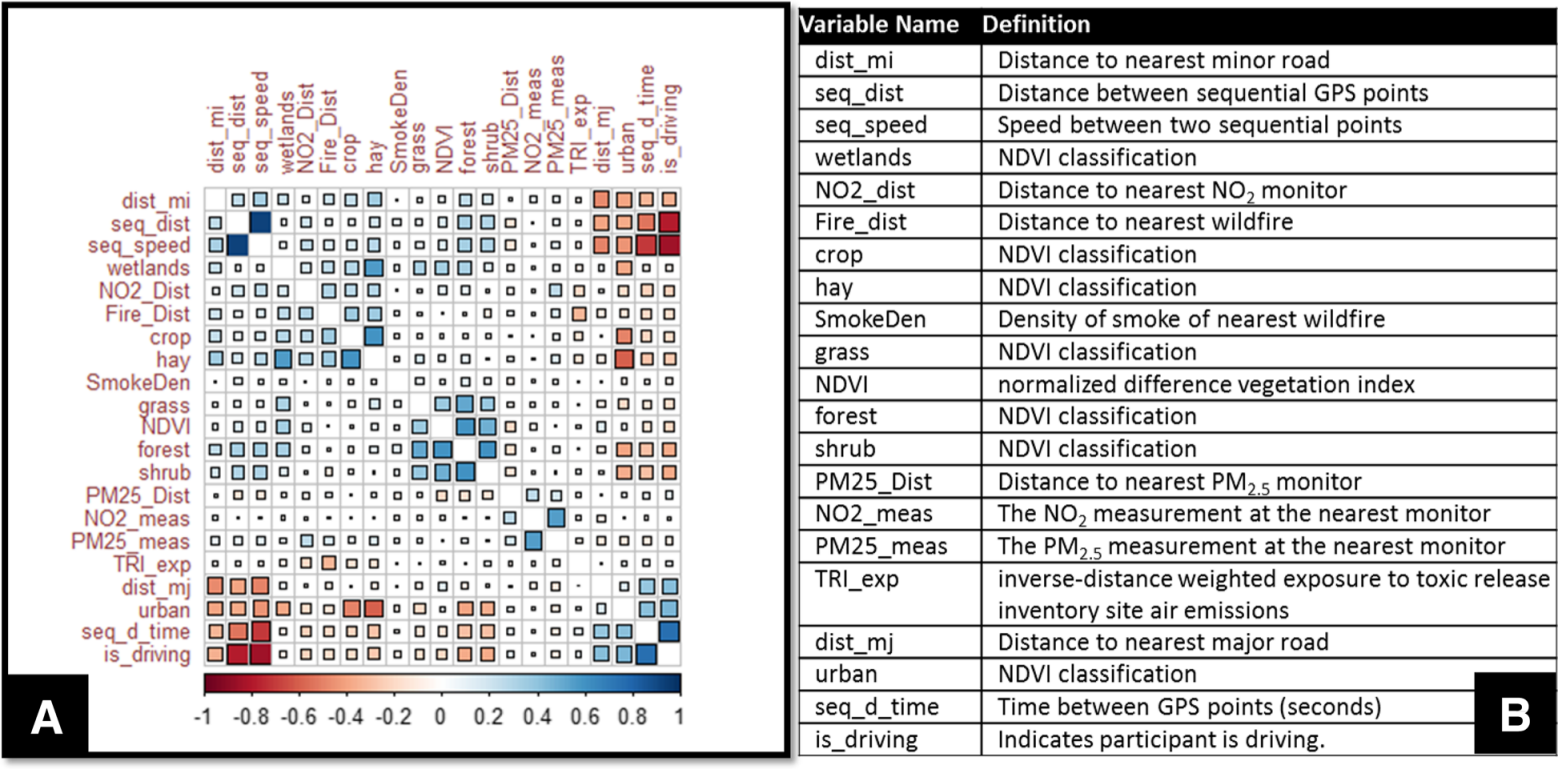
Correlations between location-derived variables. **a** Correlations are sorted to group negative (red) and positive (blue) correlations. Color intensity and the size of the squares are proportional to the correlation coefficients. **b** Definition of variable names

Correlations between all location-based exposures can be seen in Fig. 3. For example, proximity to urban areas is positively associated with time spent driving. For the majority of the study, participants spent their time in or near urban areas (Additional file 1: Table S4). Conversely, proximity to an NDVI classification of urban was negatively correlated with proximity to NDVI classifications of land used for hay or crops (Fig. 3). These associations are expected and suggest that future work may expand to evaluate correlations between TRI sites, wildfire smoke, chemical exposure and respiratory health.

**Fig. 4:**
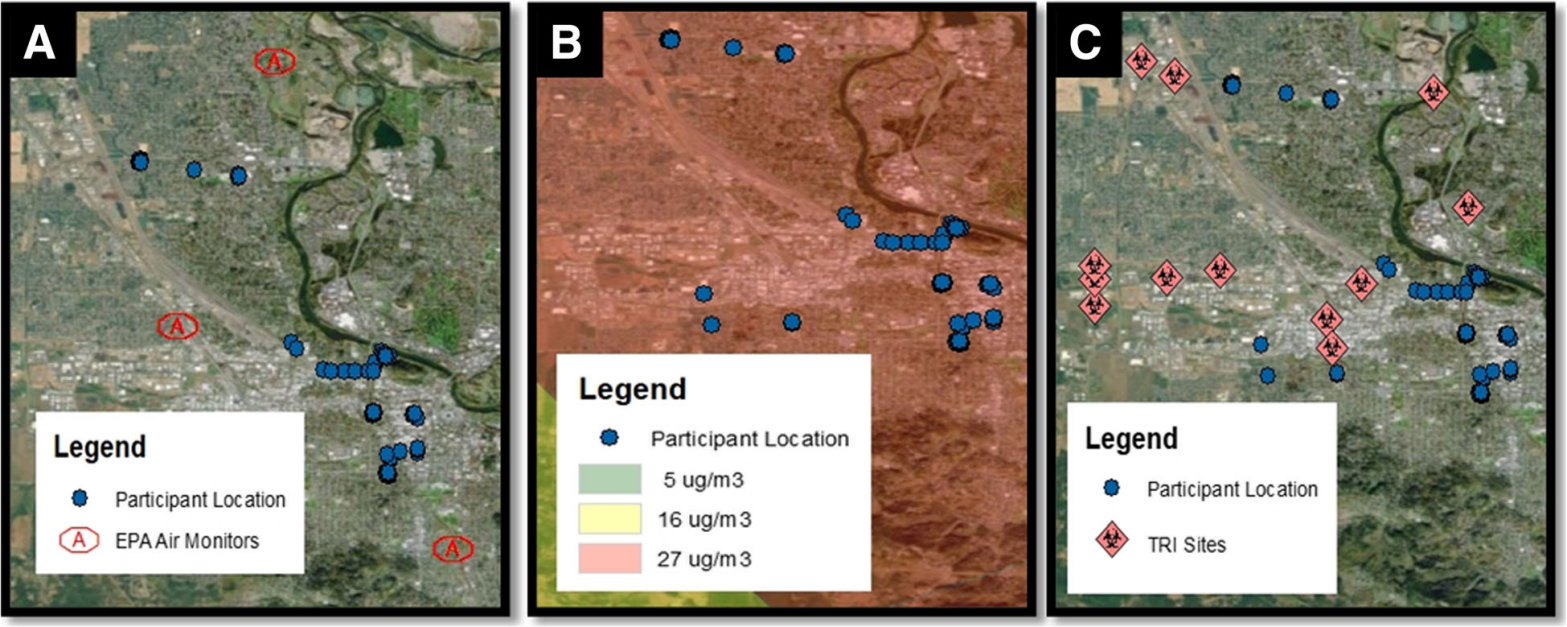
Participant location and environmental exposures. Displays the GPS locations of participants (blue dots, *n* = 4) on August 22, 2015 plotted in relationship to (**a**) EPA Air Quality Monitors (red outline), (**b**) wildfire smoke density, measured as a range of low (5 μg/m^3^) medium (16 μg/m^3^) or high (17 μg/m^3^) and (**c**) TRI sites (red diamond)

The sensitivity of the GPS data allowed for analysis of individual proximity to air quality monitors, exposure to wildfire smoke/proximity to wildfires and proximity to sources of industrial toxic releases (Fig. 4). Exposure to wildfire smoke is of increasing concern on the west coast. During the pilot phase, the nearest wildfire was over 25 km away, with participants potentially exposed to 16 μg/m^3^ of smoke daily during the data collection week (Additional file 1: Table S3).

**Fig. 5:**
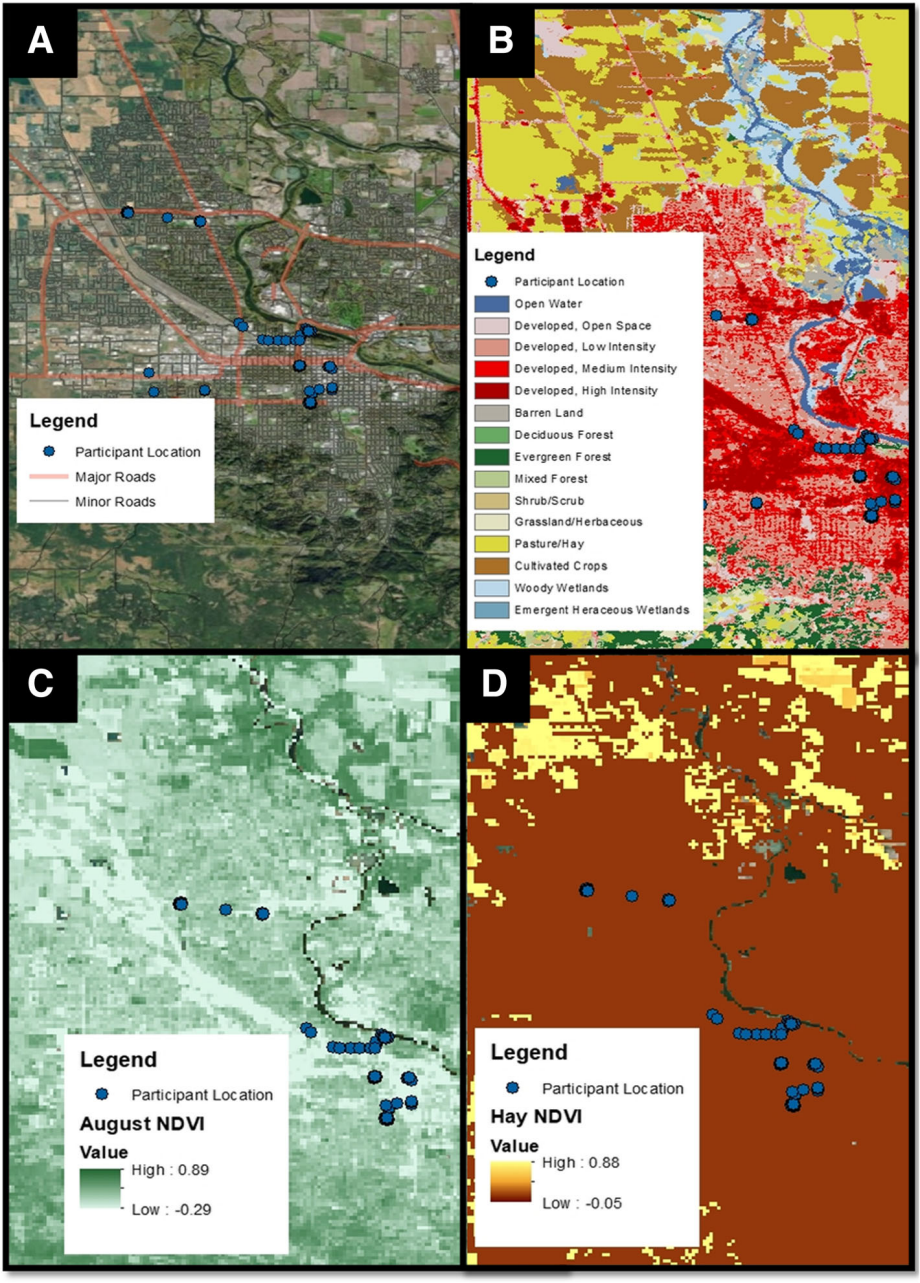
Participant proximity to roads and vegetation. Location (blue dots, n = 4) on August 22, 2015 plotted with (**a**) major and minor roads, (**b**) 2014 Oregon Land Classification, (**c**) August NDVI, and (**d**) August Hay NDVI

Use of public databases also allowed investigation of exposure to the natural environment, to include exposure to different types of vegetation based on location (Fig. 5, Additional file 1: Table S4). As noted in Tables 1, 90% of pilot participants had allergies, and 60% specifically noted a hay fever diagnosis. Of the 9 individuals with allergies, 5 noted an allergy to plants (grass, hay, trees, pollen). Therefore, evaluating proximity to known allergy-inducing vegetation may be useful.

#### 3. Reliability of the portable spirometer to collect robust data and identify changes in lung function

As shown in Table 2, participants adhered to the spirometry protocol, taking 3 readings per day (93% compliance) and collecting data that met American Thoracic Society criteria for validity (94% of readings were valid; Table 2) [36].

**Table 2:** Compliance and feasibility metrics

	Metric	Compliance
Participant Compliance	*wristband*: returned all wristbands at end of study	99%
	*wristband*: compliance and quality metrics followed	90%
	*Spiro*: 3 readings/day	93%
	*Spiro*: All recorded readings done in triplicate	100%
	*ELF Tracker*: Answered 3 questionnaires/day	94%
	*ELF Tracker*: Answered evening questionnaire	91%
	*Log*: Filled out each day	99%
	*Phone*: Carried on person all day	95%
Wristband sensitivity	PAHs detected at measureable levels each day	Table S2
	Variable chemical concentrations detected between days and between participants	See Fig. 2
Data transfer	Study ID included with all transmitted data	90%
	All data received was accurately time-stamped	100%
	No data gaps (i.e. participant indicated readings taken but PNNL received no data)	95%
	Location data received	95%
	Spirometry data received	95%
	Questionnaire data received	95%
Data Accuracy	*Spirometer*: Values met American Thoracic Society guidelines for quality data: - FEV1/FVC ratio below 1.00 - In triplicate readings, the two largest FEV1 values are within a reading are within 0.150 L of each other - In triplicate readings, the two largest FVC values are within 0.150 L of each other	94%
	*Spirometer*: Portable spirometer is sensitive enough to detect variations in lung function within-day and within the study period.	See Fig. 6
	*Spirometer:* Maintains calibration during study duration (Within 2.5% of known volume)	100%

**Fig. 6:**
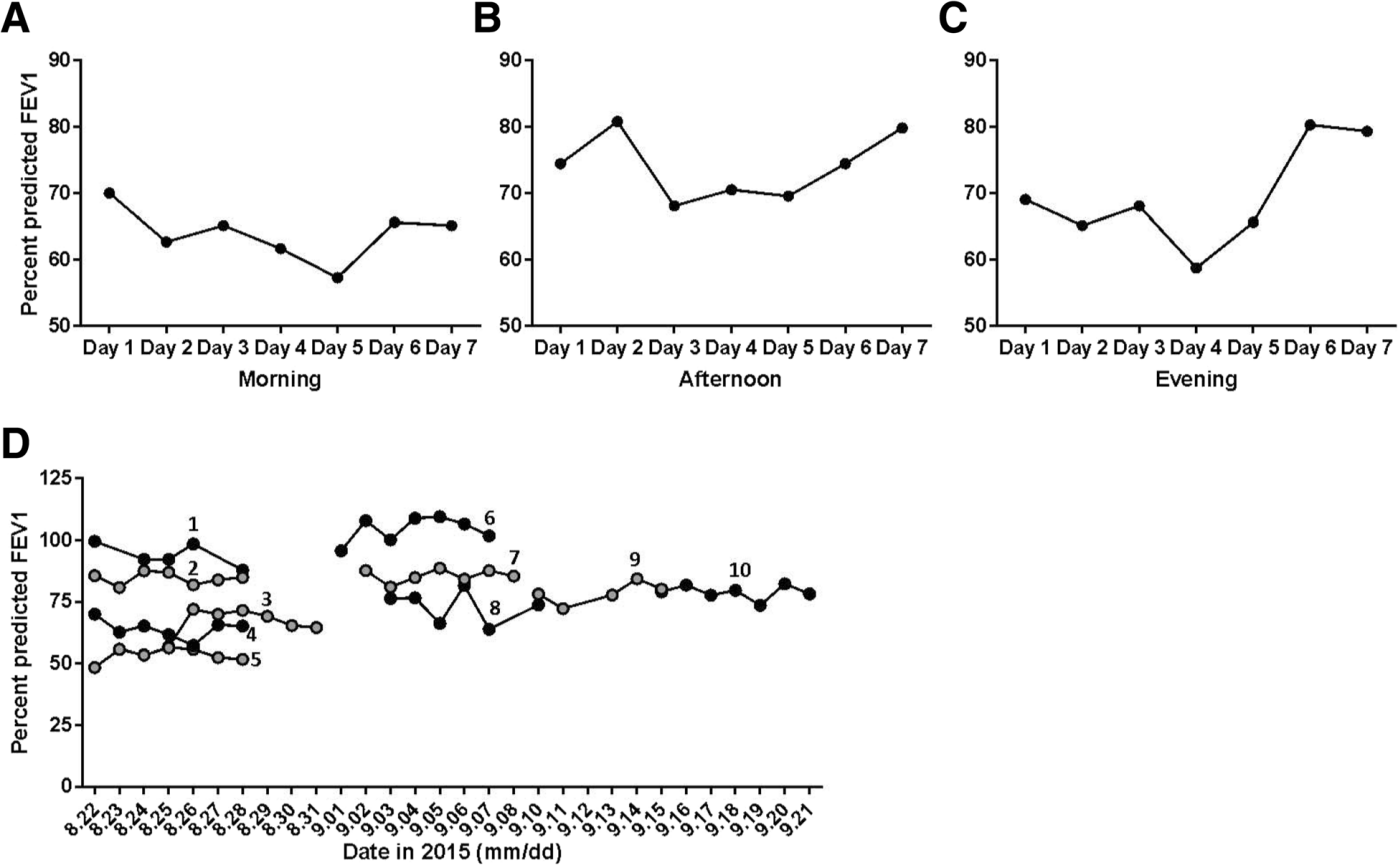
Percent predicted FEV_1_. Data for an ELF participant is shown across the seven day study period in the **a** morning, **b** afternoon and **c** evening. **d** Percent predicted FEV1 values from morning spirometry readings are shown for all ten study participants. Values are marked numerically [1–10] to delineate individual results

Changes in FEV1 were analyzed across the study period for intra- and inter-participant analysis. The morning, afternoon and evening FEV_1_ values for one representative ELF participant is shown in Fig. 6a-c. To evaluate changes in lung function between participants, the percent expected FEV_1_ was calculated. As shown in Fig. 6d, this method allows for analysis of changes in lung function across the study population.

#### 4. Data transfer within the ELF (spirometer > phone) and from the ELF to computer servers for secure storage

The ELF Tracker performed as designed (Table 2). Over 500 spirometry values were transmitted to the research servers. Of the data collected (wristband identifiers, participant identifier, time-stamps, location data, etc.), 95% were transmitted accurately and in near real-time. The lower level of compliance with data (spirometer, location and questionnaire) is due to data from one participant on the last day of the study being stored on the phone and not transmitted, equivalent to 5% of the total data transmitted within compliance. This was due to the phone either being out of service or turned off prematurely (an issue replicated in internal testing). However, as the ELF Tracker was designed to store all data locally, the data can be retrieved either directly from the phone, or by opening the App and allowing it to connect to servers automatically. The latter allowed full recovery of the data when the phone was returned upon study completion.

#### 5. Ability of participants to utilize the device for data collection with a high degree of compliance

The ELF Tracker captured real-time compliance metrics. In the evening, participants were asked questions designed to gauge compliance with basic study protocol, for example carrying the phone all day and wearing a different wristband all day. However, only 91% of the evening questionnaires were completed, making overall compliance analysis difficult (Table 2). For the days these questionnaires were completed, compliance was high, with the majority of participants reporting that they kept the phone on them all day and wore a different wristband each day.

#### Feedback on the ELF

A basic assessment of compliance was conducted in addition to specific questions regarding the use of the device (ease of using the device, wristband comfort, ease at following the protocol) and suggestions for improvement (Table 3). Participants were contacted by telephone 1–2 weeks upon completion of the study protocol. Participant feedback was largely positive regarding ability to follow the protocol and the use of the ELF as a tool.

**Table 3:** Results from follow-up interviews conducted after the 7-day study period

*n* = 10	
	*Yes*
Were you able to keep the ELF with you easily enough?	90%
Did you wear a different wristband each day?	90%
Was the wristband:	
*Comfortable?*	100%
*In your way?*	0%
*Easy to switch each day?*	90%
Did you place the wristband back in its bag and seal the bag?	100%
Did you use the spirometer three times a day?	100%
Was the cell phone application easy to use?	100%
Was the spirometer easy to use?	100%
Did you have any problems charging the cell phone or spirometer?	0%

Overall, participants suggested small changes to the labeling system used on the wristband packaging to better identify the appropriate daily wristband. Regarding the use of the ELF as a community tool, participants reported wanting different sizes of the wristband. Additional considerations centered on privacy, with participants noting it was difficult to take the spirometry measurements, especially during the day when at work. Furthermore, participants noted it was inconvenient to carry the study cell phone in addition to their personal phone.

## Discussion

This work describes the refinement and feasibility testing of a novel environmental health tool and a description and demonstration of preliminary exposure and health outcome measures. The ELF was designed to address community concerns, while also collecting robust data capable of addressing environmental health research concerns. While the use of cell phones to collect location and health symptoms/diagnostics have been successful [27–30], the ELF leverages multiple technologies to expand the capabilities afforded by a mobile phone alone. Importantly, the ELF combines environmental monitoring (i.e. detection of PAHs) with location as well as quantifiable respiratory health measures.

The data indicate that the ELF is easy to use by research participants, and capable of gathering the type of data needed to begin addressing the larger research questions around exposure and health. The high degree of compliance indicates that the tool is easy to use. The ELF Tracker can meet several of the criteria needed for community-engaged research, specifically the need for a tool that can collect robust and rigorous data that is comparable to other scientific studies [51, 52].

To our knowledge, this is the first demonstration of PAH levels in wristbands being measured concurrently with FEV_1_ levels. Personal exposure to PAHs has been previously associated with several adverse outcomes related to lung function [19, 21–25], and this easy-to-use, integrated ELF tool can be used to further explore these relationships. Using this dataset, the ELF collected chemical exposure data for up to 62 PAHs each day. The exposure data demonstrated unique patterns of exposure between individuals and identified the most common PAH exposures across individuals. Previous work has demonstrated that silicone wristbands sequester volatile and semi-volatile compounds and illustrate spatial differences between individuals [3, 16, 53–57]. Here, we detect PAHs in non-occupational 24-h deployment periods using wristbands. In larger studies with the ELF, we will be able to directly compare PAH exposure profiles from wristbands with FEV_1_ measurements from spirometers. While this feasibility study focused on PAHs, currently the wristband can be analyzed for 1530 chemicals, allowing future analysis to evaluate a larger chemical inventory [17].

The ELF Tracker collected physical location data using the smart-phone GPS capability. Regional built environment and atmospheric exposures are associated with multiple respiratory health outcomes. Acute outdoor fine particulate matter and ozone [58, 59], industrial air emissions [60], and traffic-related air pollutants [59, 61] are associated with increased rates of asthma-related hospital visits. Urban nature is also associated with respiratory health outcomes, both positive and negative. While exposure to parks, tree canopy, and gardens are associated with decreased concentrations of air pollution and decreased rates of asthma [62] and asthma-related hospitalizations [63], exposure to pollen is associated with increased rates of allergic airway inflammation [49, 64]. Evaluating these ambient environmental exposures has significant potential to reduce residual confounding and adjust for competing risks in air pollution epidemiology. The collected GPS data can therefore be used to look at multiple metrics, such as co-occurring exposure to pollutants like PM_2.5_, NO_2_ and ozone, or determine exposure from nearby emission sources, to include major roads or conversely, to estimate time spent near green spaces. Currently, such correlations are dependent on stationary air monitoring networks, such as the Air Quality Data Mart system run by the Environmental Protection Agency, or the Toxic Release Inventory [47, 65]. However, in Eugene, OR, there are only two monitors within the city, and a third located 25 miles south of the city. As a result, there are geographic and spatial gaps within the linked air quality monitor and location data. TRI data is limited temporally; the data is representative of total air emissions averaged over one year, and therefore may not be representative of exposure during the times participants were monitored. However, emerging data sources such as Google Street View can monitor the built environment [66, 67] and collect air quality measurements [68]. Growing use of the NOAA Hazard Mapping System has allowed evaluation of wildfire smoke exposure [69–71], although the system is limited by cloud cover, which can interfere with identification of smoke and/or fire. Additionally, citizen science efforts have resulted in networked community air monitors to measure urban air quality [72]. These are examples of publicly available databases that can evaluate relationships between locations and PAH sources. We show here that the ELF Tracker can be integrated with multiple databases, allowing analysis of personal location data to sources of pollution as well as time spent near sources.

The portable spirometer allowed analysis of lung function within and between participants with over 90% of all tests collecting valid spirometry data. Furthermore, over 90% of all readings complied with study protocol and accepted spirometry validity guidelines, indicating that the portable Spirotel® spirometer is easy to use, conforms to standards and collects robust, valid data. Finally, the ELF was well-received by participants and demonstrated an overall compliance rate of over 90% across all components. Furthermore, the various datasets generated by the ELF, in combination with existing datasets (NOAA Hazard Mapping, EPA Data Mart, etc.) demonstrated the feasibility of integrating multiple datasets to identify correlations between health outcomes, chemical exposures and location metrics (TRI sites, NOAA Hazard Mapping, EPA Data Mart). This allows a comprehensive evaluation of exposure and health outcomes beyond measuring a single metric of exposure. The purpose of this study was to evaluate and assess the ELF as an environmental health tool capable of being used by study participants with a high degree of compliance, collecting multiple data types (chemical exposure, physical location and respiratory health outcomes), and generating valid, accurate datasets. This trial resulted in a refinement of the ELF protocol, dropping the afternoon spirometry readings and improving the GPS sensitivity to better calculate time spent indoors vs. outdoors. While the ELF is capable of meeting the defined metrics, limitations include the small sample size (*n* = 10), preventing any exposure-health insights. Regarding the use of the ELF as an environmental health tool, we have previously conducted research evaluating the ease-of-use and compliance with the ELF tool [1], as well as the efficacy of using an online app to collect health and exposure metrics [2]. Furthermore, we have published on the use of the wristband as a tool that demonstrates high compliance [10, 56, 57]. Importantly, while the sample size is small, it generated relevant and diverse data, allowing insights into data management and data integration methodologies for use in public health.

The ELF tool is now being used in a panel study to evaluate relationships between chemical exposure, physical location and lung function. Future work will enable download of the ELF Tracker on multiple operating systems (Android, iPhone, Windows, Blackberry). The ability to utilize personal phones may improve compliance and reduce burden on the participant [30]. Finally, we have collaborated with community liaisons to begin developing an interactive, online report-back format to allow study participants to view their data (manuscript in preparation). Previous studies utilizing the wristband alone have reported data back to participants to enable improved understanding of exposure and provide mechanisms to reduce exposure [56, 57].

## Conclusions

Community-based participatory research studies are designed to address relevant and timely environmental health research questions; here, we describe a novel environmental health tool that can address complex questions around personal exposure to chemical contaminants and potential associated health outcomes. This tool has a high degree of compliance by participants and collected robust data allowing analysis of chemical exposure patterns across participants, relationship to the physical environment and relationship to respiratory health measures.

## Additional file

Additional file 1: Supplemental Tables 1-4 and Supplemental Equations 1-4. (DOCX 37 kb)

## Data Availability

The datasets used and/or analyzed during the current study are available from the corresponding author on reasonable request.

## Abbreviations

ATS: American Thoracic Society
BLOD: Below Limit Of Detection
CCV: Continuing calibration verifications
ELF: Exposure, Location and lung Function
EPA: United States Environmental Protection Agency
FEV_1_: Forced Expiratory Volume in one second
FVC: Forced Vital Capacity
GPS: Global Positioning Services
GSM: Global System for Mobile communication
IDW: Inverse Distance-Weighted
ISO: International Organization for Standardization
LIMS: Laboratory Information Management System
LOD: Limit Of Detection
N0_2_: Nitrogen Dioxide
NDVI: Landsat-based National Difference Vegetation Index
NHANES: National Health and Nutrition Examination Survey
NIU: Normalized Intensity Units
NOAA: National Oceanic and Atmospheric Administration
PAHs: Polycyclic Aromatic Hydrocarbons
PEF: Peak Expiratory Flow
PM_2.5_: Particulate Matter with a diameter of 2.5 μm
PNIU: Proportional Normalized Intensity Units
PNNL: Pacific Northwest National Laboratory
PTFE: polytetrafluoroethylene
REST-API: Representational State Transfer Application Programming Interface
SD: Standard Deviation
TRI: Toxic Release Inventory
UDOT: United States Department Of Transportation
USGS: United States Geological Survey
