# Supplementary material for: Development of an environmental health tool linking chemical exposures, physical location and lung function
Source: BMC Public Health. 2019 Jul 1;19:854. doi: 10.1186/s12889-019-7217-z (PMC6604385; doi:10.1186/s12889-019-7217-z)
Supplement: Supplementary file 1 — Supplemental Tables 1-4 and Supplemental Equations 1-4. (DOCX 37 kb) [file 12889_2019_7217_MOESM1_ESM.docx]

**Supplementary Information**

| **Database** | **Data Source** | **Website(s)** |
| --- | --- | --- |
| **Landsat 8 NDVI** | USGS  Google Earth | landsat.usgs.gov/landsat-data-access  https://explorer.earthengine.google.com/ #detail/LANDSAT%2FLC8_L1T_TOA |
| **National Land Cover Database** | USGS  Google Earth | catalog.data.gov/dataset/national-land-cover-database-nlcd-land-cover-collection  https://explorer.earthengine.google.com/ #detail/USGS%2FNLCD |
| **PM_2.5_ & NO_2_** | EPA | https://aqs.epa.gov/aqsweb/documents/ data_mart_welcome.html |
| **PM2.5 Model** | Van Donkelaar et al, 2016 | pubs.acs.org/doi/abs/10.1021/acs.est.5b05833?journalCode=esthag |
| **NO2 Model** | Larkin et al, 2017 | pubs.acs.org/doi/abs/10.1021/acs.est.7b01148 |
| **Toxic Release Inventory** | EPA | https://www.epa.gov/toxics-release-inventory-tri-program/ tri-data-and-tools |
| **Major & Minor Roads** | UDOT | [www.census.gov/geo/maps-data/data/tiger-line.html](http://www.census.gov/geo/maps-data/data/tiger-line.html) |
| **Wildfire and Wildfire Smoke** | NOAA | http://satepsanone.nesdis.noaa.gov/pub/volcano/FIRE/ HMS_ARCHIVE/2017/GIS/ |

**Table S1**. Data sources and corresponding websites for deriving location-based environmental exposures.

| PAH | LOD | Concentration (ng wristband^-1^) | | | Percent of Wristbands Detected the PAH in Concentrations Above LOD |
| --- | --- | --- | --- | --- | --- |
|  |  | Median | Minimum | Maximum |  |
| *naphthalene | 1.0 | 32 | 9.0 | 360 | 100% |
| *2-methylnaphthalene | 0.70 | 38 | 11 | 6400 | 100% |
| *1-methylnaphthalene | 0.28 | 21 | 8.6 | 4200 | 100% |
| *2-ethylnaphthalene | 0.97 | 8.8 | 3.2 | 1200 | 100% |
| *2,6-dimethylnaphthalene | 0.89 | 27 | 1.4 | 4100 | 100% |
| *1,6-dimethylnaphthalene | 0.81 | 36 | 14 | 4600 | 100% |
| 1,4-dimethylnaphthalene | 1.2 | 4.6 | BLOD | 560 | 84% |
| 1,5-dimethylnaphthalene | 1.2 | 4.2 | BLOD | 420 | 77% |
| 1,2-dimethylnaphthalene | 0.94 | 7.1 | BLOD | 17 | 71% |
| 1,8-dimethylnaphthalene | 0.83 | BLOD | BLOD | BLOD | 0% |
| 2,6-diethylnaphthalene | 0.81 | BLOD | BLOD | 82 | 2% |
| acenaphthylene | 2.3 | BLOD | BLOD | 72 | 10% |
| acenaphthene | 1.1 | 2.0 | BLOD | 420 | 50% |
| *fluorene | 0.79 | 24 | 10 | 210 | 100% |
| *dibenzothiophene | 0.24 | 6.3 | 2.3 | 16 | 100% |
| *phenanthrene | 0.46 | 58 | 24 | 140 | 100% |
| anthracene | 1.1 | BLOD | BLOD | BLOD | 0% |
| 2-methylphenanthrene | 0.39 | 18.5 | BLOD | 42 | 98% |
| 2-methylanthracene | 0.47 | BLOD | BLOD | BLOD | 0% |
| 1-methylphenanthrene | 1.1 | 12 | BLOD | 32 | 98% |
| 9-methylanthracene | 0.87 | BLOD | BLOD | BLOD | 0% |
| 3,6-dimethylphenanthrene | 0.42 | BLOD | BLOD | 10 | 10% |
| 2,3-dimethylanthracene | 0.34 | BLOD | BLOD | BLOD | 0% |
| fluoranthene | 0.54 | 9.0 | BLOD | 25 | 95% |
| 9,10-dimethylanthracene | 0.85 | BLOD | BLOD | BLOD | 0% |
| pyrene | 0.42 | 9.0 | BLOD | 28 | 94% |
| retene | 0.84 | 11 | BLOD | 47 | 90% |
| benzo[a]fluorene | 1.7 | BLOD | BLOD | BLOD | 0% |
| benzo[b]fluorene | 1.7 | BLOD | BLOD | BLOD | 0% |
| benzo[c]fluorene | 0.30 | BLOD | BLOD | 2.5 | 5% |
| 1-methylpyrene | 0.38 | BLOD | BLOD | 15 | 24% |
| benz[a]anthracene | 0.75 | BLOD | BLOD | 9.9 | 2% |
| cyclopenta[cd]pyrene | 0.53 | BLOD | BLOD | BLOD | 0% |
| triphenylene | 0.41 | BLOD | BLOD | 6.0 | 6% |
| chrysene | 0.50 | BLOD | BLOD | 9.6 | 11% |
| 6-methylchrysene | 0.89 | BLOD | BLOD | BLOD | 0% |
| 5-methylchrysene | 1.7 | BLOD | BLOD | BLOD | 0% |
| benzo[b]fluoranthene | 0.37 | BLOD | BLOD | BLOD | 0% |
| 7,12-dimethylbenz[a]anthracene | 0.94 | BLOD | BLOD | 5.0 | 2% |
| benzo[k]fluoranthene | 0.53 | BLOD | BLOD | BLOD | 0% |
| benzo[j]fluoranthene | 0.56 | BLOD | BLOD | 5.1 | 5% |
| benz[j]and[e]aceanthrylene | 1.7 | BLOD | BLOD | BLOD | 0% |
| benzo[e]pyrene | 0.71 | BLOD | BLOD | BLOD | 0% |
| benzo[a]pyrene | 1.2 | BLOD | BLOD | 6.1 | 3% |
| indeno[1,2,3-cd]pyrene | 0.26 | BLOD | BLOD | BLOD | 0% |
| dibenzo[a,h]anthracene | 1.0 | BLOD | BLOD | BLOD | 0% |
| benzo[a]chrysene | 0.74 | BLOD | BLOD | BLOD | 0% |
| benzo[ghi]perylene | 0.34 | BLOD | BLOD | 15 | 3% |
| anthanthrene | 0.33 | BLOD | BLOD | BLOD | 0% |
| naphtho[1,2-b]fluoranthene | 1.7 | BLOD | BLOD | BLOD | 0% |
| naphtho[2,3-j]fluoranthene | 1.7 | BLOD | BLOD | BLOD | 0% |
| dibenzo[a,e]fluoranthene | 0.47 | BLOD | BLOD | BLOD | 0% |
| dibenzo[a,l]pyrene | 0.48 | BLOD | BLOD | BLOD | 0% |
| naphtho[2,3-k]fluoranthene | 1.7 | BLOD | BLOD | BLOD | 0% |
| naphtho[2,3-e]pyrene | 1.7 | BLOD | BLOD | BLOD | 0% |
| dibenzo[a,e]pyrene | 6.4 | BLOD | BLOD | BLOD | 0% |
| coronene | 0.7 | BLOD | BLOD | 5.2 | 8% |
| dibenzo[e,l]pyrene | 1.7 | BLOD | BLOD | BLOD | 0% |
| naphtho[2,3-a]pyrene | 1.7 | BLOD | BLOD | BLOD | 0% |
| benzo[b]perylene | 1.7 | BLOD | BLOD | BLOD | 0% |
| dibenzo[a,i]pyrene | 1.4 | BLOD | BLOD | BLOD | 0% |
| dibenzo[a,h]pyrene | 0.52 | BLOD | BLOD | BLOD | 0% |

**Table S2.** List of target PAHs, instrumental limits of detection (LODs), and concentration results for the 62 wristbands. BLOD is reported if concentrations were below the LOD. *PAHs found in all wristbands (n = 9).

|  | **Min** | **Average** | **Max** | **SD** |
| --- | --- | --- | --- | --- |
| **Sequence Distance** (meters) | 0.00 | 445 | 2829 | 614 |
| **Sequence time** (seconds) | 126 | 664 | 985 | 215 |
| **Sequence Speed** (km/hr) | 0.00 | 347 | 16327 | 2115 |
| **Toxic Release Inventory IDW Exposure** (lbs/km^2^) | 10 | 55 | 97 | 23 |
| **NO_2_ Distance** (meters) | 52974 | 132894 | 167829 | 29514 |
| **NO_2_ Measure** (ppb) | 12 | 16 | 22 | 3.0 |
| **PM_2.5_ Distance** (meters) | 2006 | 7358 | 34058 | 6077 |
| **PM_2.5_ Measure** (ppb) | 4.0 | 6.0 | 10 | 1.0 |
| **Closest major road** (meters) | 24 | 601 | 11501 | 1473 |
| **Closet minor road** (meters) | 9.0 | 85 | 1632 | 212 |
| **Closet Wildfire** (kilometers) | 25 | 193 | 489 | 161 |
| **Wildfire Smoke Density** (ug/m^3^) | NA | 5.0 | 16 | NA |
| **Closet Wildfire Smoke** (kilometers) | 0.00 | 550 | 2099 | 632 |

**Table S3. Location and Environmental Exposures for all GPS Locations.** GPS coordinates were used to calculate proximity to point sources via the Toxic Release Inventory (TRI) database, as well as proximity to ambient air pollutants (nitrogen dioxide, PM_2.5_), roadways, wildfires and estimated exposure to wildfire smoke. IDW – Inverse distance-weighted. TRI exposure to total air emissions (sum of stack and fugitive annual air emissions) for all sites within 10km of participants. Wildfire smoke density is classified as a categorical variable with low (5μg/m3), medium (16μg/m3), and high (27μg/m3) measurements. Sequence distance is the distance between sequential GPS points. Sequence time is the amount of seconds between the sequential GPS points. Sequence speed is calculated by taking the time sequence distance and dividing it by the sequence time.

| **Vegetation** | **Min** | **Average** | **Max** | **SD** |
| --- | --- | --- | --- | --- |
| **Crop (NIU)** | 0.00 | 0.010 | 0.073 | 0.016 |
| **Crop Proportion (PNIU)** | 0.00 | 0.027 | 0.197 | 0.043 |
| **Hay (NIU)** | 0.00 | 0.027 | 0.135 | 0.038 |
| **Hay Proportion (PNIU)** | 0.00 | 0.075 | 0.423 | 0.109 |
| **Grass (NIU)** | 0.00 | 0.004 | 0.038 | 0.006 |
| **Grass Proportion (PNIU)** | 0.00 | 0.009 | 0.060 | 0.012 |
| **Forest (NIU)** | 0.00 | 0.021 | 0.297 | 0.063 |
| **Forest Proportion (PNIU)** | 0.00 | 0.035 | 0.440 | 0.097 |
| **Shrub (NIU)** | 0.00 | 0.005 | 0.086 | 0.015 |
| **Shrub Proportion (PNIU)** | 0.00 | 0.008 | 0.131 | 0.022 |
| **Urban (NIU)** | 0.00 | 0.282 | 0.406 | 0.064 |
| **Urban Proportion (PNIU)** | 0.00 | 0.889 | 1.000 | 0.200 |
| **Wetlands (NIU)** | 0.00 | 0.009 | 0.047 | 0.011 |
| **Wetlands Proportion (PNIU)** | 0.00 | 0.021 | 0.086 | 0.027 |

**Table S4. Location and Vegetation Exposure for all GPS Locations.** NDVI for land classified vegetation types were used to calculate normalized intensity units (NIU) as well as proportional normalized intensity units. Proportional normalized intensity units (PNIU) were calculated by dividing each GPS point’s buffer surface by the total amount of each land classification type.

**Supplemental Equations**

$\frac{\sum_{j=i}^{n} exposure_{i}* \Delta time_{j}}{\sum_{j=i}^{n} \Lambda time_{j}}$

Where

exposure_i_ is the exposure of interest,

Δtime_j_ is the amount of time in seconds between GPS coordinate sample j and j+1, and

n is the number of GPS coordinates in the time interval of interest

**Supplemental Equation 1.** Equation for evaluation exposure to daily and weekly PM2.5.

$\frac{\sum_{j=i}^{n} \sum_{i=i}^{m} emission_{i}/distance_{i}^{2}* \Delta time_{j}}{\sum_{j=i}^{n} \Lambda time_{j}}$

Where

emission_i_ is annual toxic release inventory estimate for site i

distance_i_ is the distance in km from site i to the GPS coordinate

Δtime_j_ is the amount of time in seconds between GPS coordinate sample j and j+1,

m is the number of TRI sites within 10km of sample j

n is last GPS coordinate sample in the time interval of interest

**Supplemental Equation 2**. Equation for calculation daily and weekly exposure to toxic release inventory emissions.

$\frac{\sum_{j=i}^{n} \sum_{i=i}^{m} road\_length_{i}* \Delta time_{j}}{\pi r^{2}*\sum_{j=i}^{n} \Lambda time_{j}}$

Where

road_length_i_ is the length of road (in meters) for road segment i

Δtime_j_ is the amount of time in seconds between GPS coordinate sample j and j+1,

m is the number of road lengths within the buffer radius of GPS coordinates for sample j

n is the number of GPS samples within the time interval of interest, and

r is the buffer radius cut-off for including roads in the summation

**Supplemental Equation 3**. Equation for calculation the time-weighted length of roads within a 100m buffer of each GPS coordinate.

$\frac{\sum_{j=i}^{n} \sum_{i=i}^{m} NDVI_{i}*Ind_{i} \Delta time_{j}}{\pi r^{2}*\sum_{j=i}^{n} \Lambda time_{j}}$

Where

NDVI_i_ is the estimated vegetation level at location i within the buffer for GPS sample j,

Indi_i_ the binary indicator of whether location i is classified as the vegetation type of interest,

Δtime_j_ is the amount of time in seconds between GPS coordinate sample j and j+1,

m is the number of locations (i.e. pixels in satellite imagery) within the buffer for GPS sample j

n is the number of GPS samples within the time interval of interest, and

r is the buffer radius (constant for all GPS samples)

**Supplemental Equation 4**. Equation for calculating weekly exposure to multiple vegetation types.
